# Editorial: Translational Insights Into Pancreatic Ductal Adenocarcinoma

**DOI:** 10.3389/fcell.2022.875836

**Published:** 2022-04-01

**Authors:** Maarten F. Bijlsma, Peter J. Bailey, Marc P. Stemmler

**Affiliations:** ^1^ Laboratory for Experimental Oncology and Radiobiology, Cancer Center Amsterdam, Center of Experimental Molecular Medicine, University of Amsterdam, Amsterdam, Netherlands; ^2^ Oncode Institute, Amsterdam, Netherlands; ^3^ Department of General Surgery, Heidelberg University Hospital, Heidelberg, Germany; ^4^ Section Surgical Research, University Clinic Heidelberg, Heidelberg, Germany; ^5^ Institute of Cancer Sciences, University of Glasgow, Glasgow, United Kingdom; ^6^ Experimental Medicine 1, Friedrich-Alexander University of Erlangen-Nürnberg, Erlangen, Germany

**Keywords:** PDAC—pancreatic ductal adenocarcinoma, organoids, tumor microenvironment, cell plasticity, cancer subtype classification, multi-omics analyses, tumor stroma, preclincal model

**FIGURE 1:**
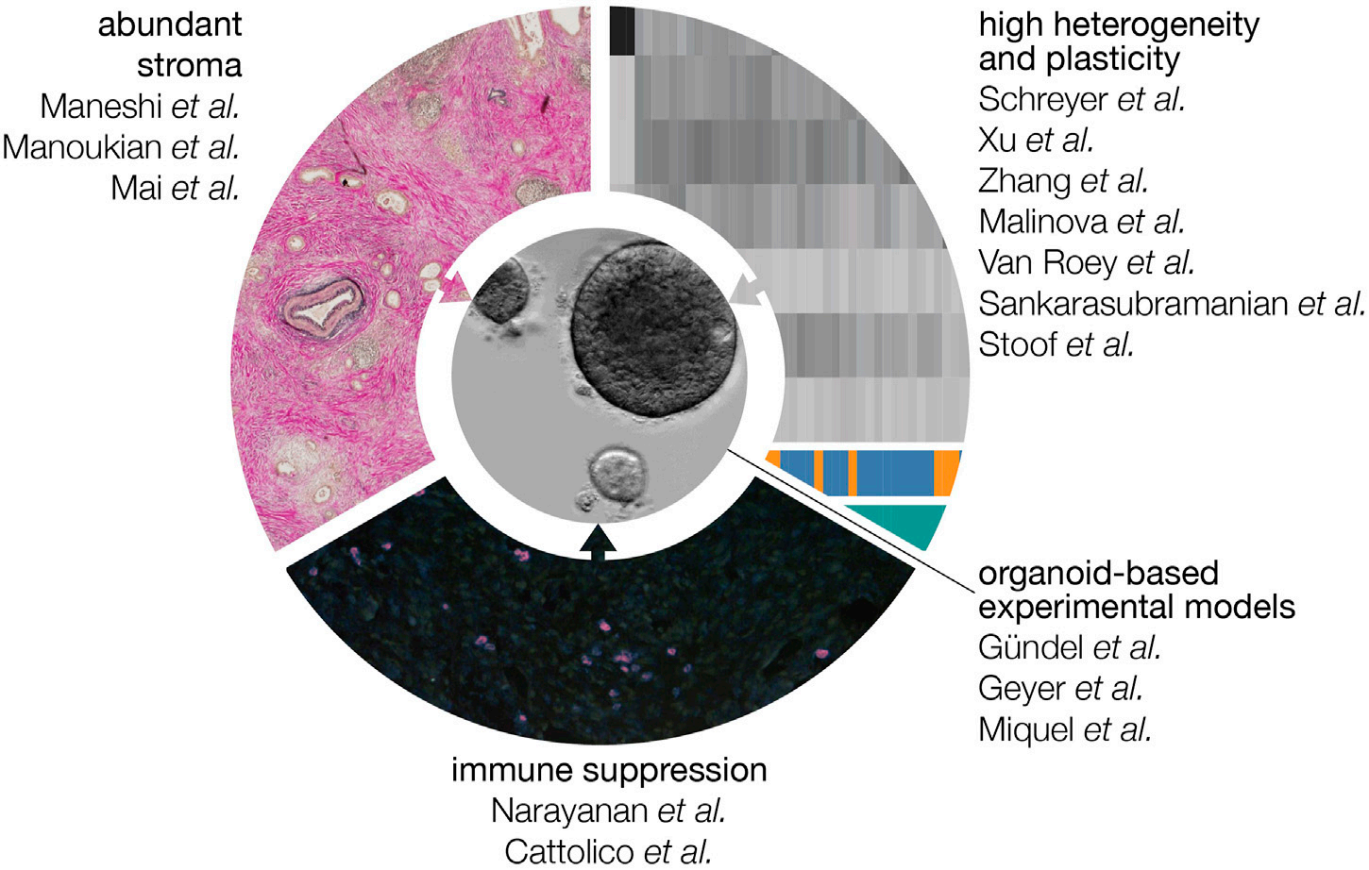
Features that contribute to the poor prognosis of PDAC. The abundant stroma, high degree of heterogeneity at the level of gene expression, and immune suppressive microenvironment all contribute to a tumor type that quickly progresses to an incurable stage and is resistant to currently available regimens. In this Frontiers topic, the contributors explain how the organoid culturing system can be used and modified to address these tumor-promoting features experimentally.

Pancreatic ductal adenocarcinoma (PDAC) carries the worst prognosis of all cancer types, and its incidence is increasing year on year. Currently available treatment options in both the resectable setting as well as inoperable disease are insufficiently effective. In this Frontiers Topic, members of the European Commission-funded PRECODE consortium and the wider pancreatic research community, provide their views on several key features of PDAC that contribute to its poor outcome. These include the abundant collective of non-tumor cells and material known as the stroma, the immune landscape unique to this disease, and intrinsic molecular subtypes that exhibit distinct neoplastic characteristics and plasticity in response to both stromal cues and therapy (Figure 1). A major goal of the PRECODE consortium was the establishment of a well characterized pancreatic organoid resource for the study of PDAC pathobiology and the development of much-needed novel therapies. The reviews in this topic support this goal and provide detailed commentaries on the application of organotypic pre-clinical models for the study of PDAC biology and the development of next generation treatments and decision-making tools. This Frontiers topic issue contains 14 reviews and one original research article.

Despite being driven by a relatively small number of recurrent driver mutations, PDAC is characterized by distinct *heterogeneity* at the gene expression level (Figure 1). It is now clear that distinct subgroups can be identified, and that these differ mostly in the degree to which tumor cells have acquired a motile, resistant phenotype that is called *mesenchymal*. Schreyer et al. and colleagues describe the currently available array of biomarkers and omics technologies, and the limitations that stand in the way of clinical implementation such as patient selection. Xu et al. further review the clinical relevance of molecular profiling and how to achieve effective clinical translation. In an original article Zhang et al. and colleagues introduce a 5-gene TP53 mutation-associated gene signature helpful to optimize prognostic stratification. Further adding to the contributions of heterogeneity and subtypes to poor therapy responses is the readiness with which PDAC cells transition between cell states (and at the tissue level; subtypes). This plasticity also applies to the origins of the disease: it is suspected that pancreatic ductal carcinomas find their origin in acinar cells that have transitioned to a ductal fate. The reviews by Malinova et al., van Roey et al., and Sankarasubramanian et al. provide an overview of the mechanisms currently known to govern lineage infidelity and phenotype switching, and how these may contribute to drug resistance. The signaling cascades, metabolic factors, transcription factor networks, and epigenetic gene regulation that contribute to plasticity, epithelial-mesenchymal transition (EMT) and disease progression are explained. Highly related to the molecular subtypes that may associate with response is the subgroup of PDAC tumors that are defective for DNA damage repair. Stoof et al. describe how this deficiency can be leveraged to design and select for therapeutic strategies.

The sheer abundance of non-tumor cells and material known collectively as the *stroma* is known to impact tumor progression and therapy response but the exact outcomes and mechanisms remain elusive. The review by Maneshi et al. summarizes how extracellular matrix deposition and the resultant mechanical properties of the stroma impact on signaling pathways and subsequent tumor biology. The origins of the most abundant cell type in the stroma, the cancer-associated fibroblasts (CAFs) are a topic of debate. Likewise, a high degree of heterogeneity has been described to exist in this cell population that impacts on disease progression and therapy response. Manoukian et al. describes these different subsets of fibroblasts in PDAC, and how this relates to the suspected cellular origins of CAFs. Recently, another non-tumor cell entity, platelets, has been found to strongly interact with tumor cells beyond their well known roles in coagulation. For instance, they are a rich tumor biomarker matrix but also impact on tumor biology. Mai and Inkielewicz-Stepniak colleagues describe the contributions of platelets to pancreatic cancer and how these drive tumor progression.

In many tumor types, immune therapies such as checkpoint inhibitors have demonstrated promising efficacy in the clinic. In PDAC, this has not yet been the case. The abundant stroma explained above is suspected to contribute to this, as may additional features specific to PDAC. In the review by Narayanan et al. properties that allow PDAC tumors (for instance the above-mentioned low mutational burden) to evade immune surveillance are discussed in depth. Cattolico et al. further explain why the immune response is ineffective, despite detection of features in PDAC that should activate the immune system. The focus in this review is on the role of interferons in tumor-immune crosstalk, how PDAC cells bypass this signaling, and how to reactivate this signaling to improve therapeutic outcome.

Given the above considerations, the question arises how to design experiments that will give meaningful answers and allow the development and testing of therapeutic options that are effective despite the challenges specific to PDAC. Which model systems are required and appropriate, and how should they be modified to suit the specific research question? Most authors in this Frontiers topic propose that the organoid system is a solid basis from which to develop such models, and that plasticity can be faithfully captured in these cultures. In the case that contributions of the stroma are to be studied, relevant stromal cell types can be incorporated (Figure 1). This should be done in a fashion that does not perturb, for instance, their phenotype or orientation relative to the tumor cells as seen in patient tumors. A comprehensive summary about existing preclinical *in vitro* and *ex vivo* models is given by Gündel et al. and colleagues. Geyer and Queiroz explain how organ-on-chip technologies can further improve co-culture models for PDAC by more accurately modeling the physicochemical properties of PDAC. Miquel et al. make a case for the development of better models for metastatic PDAC, and how these could be developed from existing technologies.

In conclusion, this Frontiers topic issue summarizes the current challenges of PDAC treatment, pointing out that there is an urgent need to establish proper models to identify novel vulnerabilities of this devastating disease. Organotypic pre-clinical models of PDAC provide a tractable model system for understanding the dynamic interplay between neoplastic and stromal cell types. The co-culturing of genomically characterized pancreatic organoids with cell types that are constituents of the PDAC tumor microenvironment promises new insights into the pathobiology of PDAC. These approaches will also drive the development of next generation treatment options that target key tumor-stroma interactions and/or modify neoplastic programs that effectively augment systemic chemotherapy.

